# Prevalence of Antibodies against Avian Influenza A (H5N1) Virus among Cullers and Poultry Workers in Ho Chi Minh City, 2005

**DOI:** 10.1371/journal.pone.0007948

**Published:** 2009-11-23

**Authors:** Constance Schultsz, Nguyen Van Dung, Le Thanh Hai, Do Quang Ha, J. S. Malik Peiris, Wilina Lim, Jean-Michel Garcia, Nguyen Dac Tho, Nguyen Thi Hoang Lan, Huynh Huu Tho, Phan Xuan Thao, H. Rogier van Doorn, Nguyen Van Vinh Chau, Jeremy Farrar, Menno D. de Jong

**Affiliations:** 1 Oxford University Clinical Research Unit, Hospital for Tropical Diseases, Ho Chi Minh City, Vietnam; 2 Centre for Tropical Medicine, Department of Clinical Medicine, University of Oxford, Oxford, United Kingdom; 3 Centre for Poverty Related and Communicable Diseases, Academic Medical Centre, Amsterdam, The Netherlands; 4 Subdepartment of Animal Health, Ho Chi Minh City, Vietnam; 5 Preventive Medicine Center, Ho Chi Minh City, Vietnam; 6 University of Hong Kong, Hong Kong Special Administrative Region, China; 7 Hong Kong Department of Health, Hong Kong Special Administrative Region, China; 8 HKU-Pasteur Research Centre, Hong Kong Special Administrative Region, China; 9 Department of Medical Microbiology, Academic Medical Centre, Amsterdam, The Netherlands; Karolinska Institutet, Sweden

## Abstract

**Background:**

Between 2003 and 2005, highly pathogenic avian influenza A (H5N1) viruses caused large scale outbreaks in poultry in the Ho Chi Minh City area in Vietnam. We studied the prevalence of antibodies against H5N1 in poultry workers and cullers who were active in the program in Ho Chi Minh City in 2004 and 2005.

**Methodology/Principal Findings:**

Single sera from 500 poultry workers and poultry cullers exposed to infected birds were tested for antibodies to avian influenza H5N1, using microneutralization assays and hemagglutination inhibition assay with horse blood. All sera tested negative using microneutralization tests. Three samples showed a 1∶80 titer in the hemagglutination inhibition assay.

**Conclusions/Significance:**

This study provides additional support for the low transmissibility of clade 1 H5N1 to humans, but limited transmission to highly exposed persons cannot be excluded given the presence of low antibody titers in some individuals.

## Introduction

Since their re-emergence in late 2003, highly pathogenic avian influenza A (H5N1) viruses have spread across the globe, reaching endemic levels amongst poultry in several countries. The continuing occurrence of sporadic human H5N1 infections has ignited worldwide concern about an imminent influenza pandemic with potentially devastating consequences, especially if a pandemic H5N1 virus would keep its current virulence in humans. This threat persists despite the emergence of pandemic H1N1 in early 2009, either through direct adaption of H5N1 to efficient human transmission, or through reassortment with the novel H1N1 virus in swine or in humans.

Based on reported cases, the mortality of human H5N1 infections still exceeds 60% with most patients dying of rapidly progressive respiratory failure. The occurrence of mildly symptomatic and asymptomatic human H5N1 infections has been suggested by seroepidemiological studies after the 1997 H5N1 outbreak in Hong Kong [1], but a limited number of serological studies in individuals exposed to H5N1-infected patients or poultry since 2003 suggest that mild or asymptomatic human infections are rare [2], [3], [4], [5]. While these studies suggest inefficient transmission of current H5N1 viruses to humans, additional studies in individuals who are highly exposed to infected birds are essential to determine the full clinical spectrum of human H5N1 infections and assess the pandemic risk.

Vietnam has been one of the countries hit hardest by influenza H5N1 with 111 human infections reported since 2003. Beginning in early 2004, culling programs were initiated in Vietnam to contain spread of H5N1 across poultry farms. These programs identified infected poultry and provided in culling of all poultry on farms where infected poultry was found. We studied the prevalence of antibodies against H5N1 in poultry workers and cullers who were active in the program in Ho Chi Minh City in 2004 and 2005 when large scale poultry outbreaks were occurring in and around the city.

## Materials and Methods

### Survey site

The sub-department of Animal Health HCMC (AH) performed active surveillance in poultry farms (any size) in Ho Chi Minh City (HCMC), to identify and subsequently cull ducks and chickens with H5N1 infection, between October 2004 and June 2005. During this period, clade 1 H5N1 viruses were circulating in southern Vietnam [6]. District veterinary health care workers visited all poultry farms in 4 districts of HCMC, to perform serological analysis on randomly collected serum samples from ducks and chickens. These 4 districts covered all poultry farms in the HCMC area at the time of the survey. In addition, farms and households with dying poultry were identified. Positive sites were defined as farms or households with at least one sample obtained from poultry (chicken and/or ducks) positive by hemagglutination inhibition (HI) test. On these sites, all poultry was culled directly following a positive HI result, by cullers employed by AH. Poultry vaccination was not performed before or during the study period in the HCMC area.

### Enrollment

Poultry farmers were identified by AH as having worked on a farm where poultry tested H5N1 positive during the 6 months prior to April 2005. Adults (>15 years old) living or working in affected sites at least 1 week prior to identification of H5N1 infected poultry, were included in the study. All cullers who were actively involved in culling during the period December 2004–June 2005, were identified by AH. All poultry workers on positive sites and all cullers were visited by staff from the Institute of Preventive Medicine from Ho Chi Minh City in June and July 2005. Witnessed oral informed consent was obtained from all participants. The study and the consent procedure were approved by the Hospital for Tropical Diseases Ethical and Scientific Committee and the University of Oxford Tropical Research Ethical Committee.

### Questionnaire

A questionnaire was made in English and translated into Vietnamese. Participants were asked about demographic characteristics, exposure to poultry, the use of protective gear during exposure, and the occurrence of clinical symptoms during the period of study.

### Sample collection

Serum samples from poultry were collected on site and directly transferred to the laboratory of AH for analysis. Samples were tested within 48 hours of collection.

A single 5 ml blood sample was obtained from all participants in June or July 2005, transported to the laboratory of the Oxford University Clinical Research Unit (OUCRU) in HCMC, and, after separation of serum, stored in aliquots at −20°C. Aliquots of serum were sent to Hong Kong (MP and WL) on dry ice for analysis. As the time between start of poultry culling and serum sample collection was more than 3 months for all participants, only single serum samples were collected and tested.

### Laboratory testing

Serum samples from poultry were analyzed by HI as described in the Manual of Diagnostic Tests and Vaccines for Terrestrial Animals of the World Organization of Animal Health (www.oie.int/eng/normes/mmanual/a_00037.htm) using chicken red blood cells and H5N1 antigen derived from strain A\Ck\Scot\59 (provided by OIE reference laboratory VLA Weybridge, Surrey, UK). Samples from dead poultry were subjected to real-time RT-PCR for detection of the H5 gene [7].

Human serum samples were tested using three serological assays. All samples were tested by microneutralization assay using Influenza A H5N1 strain A/Vietnam/1194/2004 (Z5) (clade 1) and A/Vietnam/30850/05 (clade 2.3.4) with suppression of viral cytopathic effect as the end point [7]. In addition, all samples were tested by microneutralization assay, using Influenza A H5N1 strains A/Vietnam/3212/2004 (clade 1), with suppression of virus antigen expression as assessed by ELISA assay as endpoint, as described elsewhere [8]. Samples which were positive in this latter assay, were analyzed for cross-reactivity to other influenza A viruses by adsorption tests. Patient's serum was treated with high concentration of inactivated H1N1 and H3N2 viruses and incubated at room temperature for one hour. Treated and untreated sera were tested against H1N1, H3N2 and H5N1 viruses by the ELISA based micro-neutralization test. Four fold or greater reduction of H5 titre in treated sera indicated cross-reactivity. Single sera with a titer of 1∶80 or more were considered positive in both tests.

Human sera which tested positive at any dilution in at least one of the two tests, were retested by HI using horse red blood cells [9] and by microneutralization test with suppression of viral cytopathic effect as the end point, at the virology laboratory of OUCRU, using a clinical isolate of influenza A (H5N1) virus (A/VN/CL26/2004) representing the circulating virus at the time of the study.

## Results

### Sites

A total of 65 positive sites were identified. Of these, one site had dying chicken which were confirmed by RT-PCR to be infected with H5N1. Two additional sites had dying chicken which could not be tested for the presence of H5N1 as they had been destroyed. All other sites had ducks with positive HI tests. The number of poultry on a given site varied from 2 to 1600. The number of samples tested per site varied between 1 and 31 and the number of HI positive samples per site varied between 1 and 30.

### Poultry workers

A total of 183 poultry workers were included in the study. 89 (50%) were male (sex was not recorded for 5 poultry workers) and the median age was 36 years (range 15–78 years, age missing for 2 poultry workers). The majority of infected sites contained ducks (Table 1). Almost all poultry workers were exposed to infected poultry which had been present on the farm for at least two weeks (Table 1). Few poultry workers reported the use of protective gear during their work (Table 2). The median time between serum sample collection and culling of infected poultry was 164 days (range 134–262 days, culling date not recorded for 5 sites). None of the poultry workers showed a positive titer in any of the different tests used for determination of antibodies against H5N1.

**Table 1:** Exposure to poultry for poultry workers in the 12 months prior to serum sample collection.

	No. of poultry workers (%) N = 183
Type of animals reported on site in previous 12 months	
Chicken	101 (55.2)
Ducks/geese	177 (96.7)
Other birds	11 (6.0)
Pigs	48 (26.2)
Dying chicken reported on site in last 12 months*	
>3 months ago	32 (17.1)
< = 3 months ago	4 (2.2)
Duration of stay of culled poultry on site before culling#	
>3 months	102 (56.3)
2 weeks–3 months	77 (42.5)
<2 weeks	2 (1.1)

*Missing data for 8 poultry workers.

#Missing data for 2 poultry workers.

**Table 2:** Protective measures taken by poultry workers and cullers during live poultry exposure as part of poultry work (poultry farmers) or culling (cullers).

	*Use of protective measures during poultry exposure in 6 months prior to sample collection*	
*Protection*	poultry farmers N = 138* n (%)	cullers N = 240* n (%)
Goggles	5 (3.6)	88 (36.7)
Gown or coverall	5 (3.6)	114 (47.5)
Apron	0	7 (2.9)
Gloves	8 (5.8)	213 (88.8)
Boots	5 (3.6)	95 (39.6)
Mask, cloth	7 (5.1)	33 (13.8)
Mask, paper	10 (7.2)	181 (75.4)
Mask, N95	0	20 (8.3)

*138/183 poultry farmers and 240/317 cullers were exposed to live poultry during the 6 months prior to sample collection.

### Cullers

317 cullers were involved in culling during the period December 2004–June 2005. 195 (61.7%) were male (sex was not recorded for 1 culler) and the median age was 42 years (range 22–58 years). The median time between the last exposure and serum sample collection was difficult to assess as most cullers were involved in culling of infected poultry during extended periods of time (Table 3). The predominant culling method was the catching of live poultry in bags followed by suffocation (Table 3). All serum samples were negative by microneutralization assay which uses suppression of virus antigen expression as endpoint, using the recommended cut off values of ≥1∶80, except for two samples with titers of 1∶80 and 1∶350. However these samples turned negative after adsorption with H1 antigen. In a neutralization assay which uses suppression of viral cytopathic effect as end point, two samples showed a 1∶10 titer and one sample showed a 1∶80 titer. These three samples were subsequently tested by HI using horse blood and neutralization assay at OUCRU and showed a HI titer of 1∶80, whilst neutralization titers were 1∶20, 1∶40, and 1∶200 respectively (Table 4). An HI titer of 1∶80 is one dilution below the cut-off titer of 1∶160 as recommended by the World Health Organization [9]. The three cullers were two men and one woman, aged 26, 42 and 49 years old. All three cullers had been involved in culling during more than a year (all time periods depicted in Table 3). They did not have poultry at home or in the neighbourhood where they lived. Two of the cullers used only gloves and paper or cloth masks during culling, one culler also used a coverall and boots in addition to gloves and paper mask. None of the cullers reported symptoms during the month prior to sample collection.

**Table 3:** Exposure to poultry for cullers in the 12 months prior to serum sample collection.

	No. of cullers (%) N = 317
Keep poultry* at home	14 (4.4)
Neighbours keep poultry* at home	64 (20.4)
Regularly visit live poultry markets	163 (51.7)
Involved in cock fights	33 (10.6)
Time period when involved in culling	
December 2003 – February 2004	236 (74.4)
March – July 2004	214 (67.5)
August – November 2004	220 (69.4)
December 2004 – February 2005	282 (89)
March – June 2005	206 (65)
Culling methods used	
Catch poultry in bags	285 (89.9)
Burn poultry	105 (33.1)
Bury poultry	89 (28.1)

*chicken and/or ducks.

**Table 4:** Titers of serum samples reactive in neutralization tests and hemaglutination inhibition test (for details see Materials and Methods).

	titer			
Serum nr	NT1*	NT2**	NT3#	HI§
1	<1∶10	1∶10	1∶20	1∶80
2	<1∶10	1∶10	1∶40	1∶80
3	<1∶10	1∶80	1∶200	1∶80

*microneutralization assay using antigen expression read out.

**neutralization assay using cytopathic effect read out.

# neutralization assay using cytopathic effect read out and circulating influenza A H5N1 virus.

§ HI: hemagglutination inhibition assay using horse blood.

## Discussion

We studied the prevalence of antibodies against H5N1 among 500 poultry workers and cullers in the HCMC area in 2005. All poultry workers and cullers had a history of exposure to poultry with evidence of symptomatic infection (dying chicken with positive RT-PCR) and/or asymptomatic infection (HI positive ducks) with H5N1. None of the poultry workers or cullers had antibody titres ≥80 in microneutralization tests, which is the recommended threshold for serological evidence of H5N1 virus infection, indicating that transmission of H5N1 was low. These results are in agreement with studies performed amongst poultry workers and persons exposed to infected poultry in the household in Nigeria, Thailand and Cambodia [2], [3], [4], [5].

Interestingly, three cullers showed positive antibody responses in one of the neutralization assays, in two cases below-, and in a third case at the lower threshold of recommended cut-off values. These neutralizing antibody responses were confirmed in a different laboratory using a clinical isolate representative of the circulating virus at the time of the study. According to current guidelines, a second serological test, in this case HI using horse blood, was performed on all samples showing a titer in one of the neutralization assays. HI antibodies were observed in these three sera, albeit at titers one dilution below recommended cut-off values for confirmatory testing [9]. Thus, while none of the 500 exposed poultry workers and cullers met recommended serological criteria for H5N1 infection, our results suggest that 3 cullers may have been infected subclinically. Similar low titered antibody responses have been found in poultry farmers in Thailand [2]. Whilst the possibility exists that asymptomatic infection is associated with low antibody titers, other possible explanations for the observed low antibody response in subclinically infected individuals include the decay of antibodies over time and the use of variant viruses in the neutralization assays which do not fully match the circulating and infecting viruses across the study population. Alternatively, the antibody responses may represent cross-reactivity with circulating antibodies against other (low pathogenic) avian influenza viruses or seasonal human influenza viruses [2].

The median time between the last exposure and serum sample collection for cullers was difficult to assess as most cullers were involved in culling of infected poultry during extended periods of time (Table 3). This prolonged involvement in culling however, suggests that exposure was continuous and therefore that waning of antibody levels, if these antibodies were produced during asymptomatic infection, may be less likely. Waning of antibodies does occur however, as was shown for subclinically infected individuals in Cambodia [5]. The viruses used in neutralization assays all belonged to clade 1 H5N1 viruses which were prevalent in southern Vietnam at the time of the study [6]. Therefore, mismatch between viruses used in the neutralization assay and those which potentially infected poultry farmers or cullers seems an unlikely explanation for the observed low antibody responses.

Approximately 3% of pre-vaccine sera of US participants in an H5N1 vaccine trial had H5N1 neutralizing antibody in the microneutralization test and in HI assay using horse red blood cells [10]. These rates of sero-positivity in a population not expected to be exposed to highly pathogenic H5N1, or even low pathogenic H5-viruses, were similar to those observed in the poultry cullers in our study, which may suggest that the low positive antibody responses in our study group do not represent recent (asymptomatic) infection but more likely represent cross-reactivity with antibodies against other influenza viruses.

The exact exposures of poultry farmers and cullers are difficult to assess. Dying chicken in an area and time where H5N1 is highly prevalent, is highly indicative of infection in chicken, and therefore of exposure to those involved in farming and culling on farms where chicken were dying. However, the exposure is less clear for individuals who were exposed to infected ducks only. Whilst in the early stages of the current H5N1 outbreak infected ducks were reported dying, the virus adapted rapidly to become asymptomatic in ducks [11], [12]. However, asymptomatic ducks can shed large numbers of virus particles from the oral cavity and cloaca [11], [12]. In addition, it has been shown that the virus can be isolated from duck feathers during a longer period of time than from oropharyngeal or cloacal swabs, from asymptomatic domestic ducks [13]. The majority of cullers (90%) used culling methods which included catching live poultry in bags, and therefore exposure to high concentrations of H5N1 viruses during culling of infected ducks is likely.

In conclusion, whilst our study provides additional support for the low transmissibility of highly pathogenic clade 1 H5N1 viruses to humans, limited transmission to highly exposed persons cannot be fully excluded given the presence of low antibody titers in some individuals.
